# The Impact of Smoking on Outcomes Following Anterior Cruciate Ligament Reconstruction: A Systematic Review and Meta-Analysis

**DOI:** 10.7759/cureus.96765

**Published:** 2025-11-13

**Authors:** Abdelrahman Ibrahim, Musab Al-Musabi, Rakan Kabariti, Swarna Kempe-Gowda, Roger Wade

**Affiliations:** 1 Trauma and Orthopaedics, Princess Royal Hospital NHS Trust, Telford, GBR; 2 Trauma and Orthopaedics, University Hospitals of North Midlands NHS Trust, Stoke on Trent, GBR; 3 General Practice, University Hospitals of North Midlands NHS Trust, Stoke on Trent, GBR

**Keywords:** anterior cruciate ligament (acl) reconstruction, cigarette smoking, functional outcome of acl reconstruction, meniscus tear, meta-analysis, surgical site infection(ssi), systematic review and meta-analysis

## Abstract

The influence of smoking on postoperative outcomes following anterior cruciate ligament (ACL) reconstruction is a topic of ongoing scientific discussion and uncertainty. We aimed to conduct a systematic review and meta-analysis to compare the outcomes between smokers and non-smokers undergoing this procedure. We conducted a systematic search of electronic information sources, including MEDLINE, EMBASE, CINAHL, CENTRAL, ClinicalTrials.gov, and bibliographic reference lists. We applied a combination of free-text search and controlled vocabulary search adapted to thesaurus headings, search operators, and limits in each of the above-mentioned databases. Primary outcome parameters included surgical site infections, ACL graft rupture, revision rates, and patient-reported outcome measures (PROMs). We identified 24 comparative studies, including a total of 672,241 patients, of whom 69,113 were in the smoker group and 603,128 were in the non-smoker group. The analysis revealed that smoking was associated with a significantly higher risk of surgical site infections (OR 1.40, P=0.01). Smokers also reported significantly worse PROMs on the International Knee Documentation Committee (IKDC) score (MD -5.38, P<0.00001) and multiple Knee Injury and Osteoarthritis Outcome Score (KOOS) subscales. There was no statistically significant difference between the two cohorts for ACL graft rupture or all-cause revision rates. Smoking appears to be associated with a higher risk of surgical site infections following ACL reconstruction and is linked to significantly poorer functional PROMs.

## Introduction and background

Anterior cruciate ligament reconstruction (ACLR) is one of the most common orthopaedic procedures, with an estimated incidence of 43.5 per 100,000 people in the United States and 40.7 per 100,000 people in Sweden [1,2]. In patients with functional impairment, ACLR is the gold standard treatment intended to restore full knee stability and functionality [3].

Although ACLR is generally a safe and effective procedure, serious complications, such as septic arthritis (SA) and graft failure, can occur, resulting in prolonged rehabilitation, poorer outcomes, and the need for repeated surgery [4]. The reported incidence of postoperative SA after ACLR ranges from 0.58% to 1.8% [5-7].

Given that not all patients have an optimal outcome, the identification of modifiable predictors is critical to provide interventions that improve results and aid physicians in counselling patients regarding their prognosis [8,9]. Among various patient-related factors, tobacco use has been identified as a significant risk factor that may influence postoperative outcomes [10].

Biologically, smoking is associated with impaired wound healing and immunosuppression, as it is known to delay cell migration and decrease Type 1 collagen synthesis [9,10]. However, the existing literature investigating the impact of smoking is characterised by conflicting reports [11]. This uncertainty is challenging for clinicians, as many prior studies are limited by small sample sizes and a failure to control for the large number of covariates known to influence ACLR graft rupture and other outcomes, particularly patient-reported outcome measures (PROMs) [11,12].

These PROMs are crucial for assessing a patient's perspective on their recovery. The Knee Injury and Osteoarthritis Outcome Score (KOOS) is a patient-reported questionnaire for an athletically active population, with scores transformed to a 0-100 scale where 100 is the best possible score [8]. The IKDC Subjective Knee Form is a similar patient questionnaire evaluating knee symptoms, function, activity levels, and the ability to perform daily living tasks and sports, also scored from 0-100, with higher scores indicating better function [9]. To date, a comprehensive meta-analysis of comparative outcomes, including these critical PROMs, as well as a comprehensive meta-analysis of comparative outcomes does not exist.

Therefore, the purpose of this study was to conduct a comprehensive systematic review and meta-analysis to investigate the impact of smoking on the clinical outcomes of ACLR.

The Appendices present a graphical abstract of the impact of smoking on ACL reconstruction outcomes.

## Review

Methods

**Figure 1:**
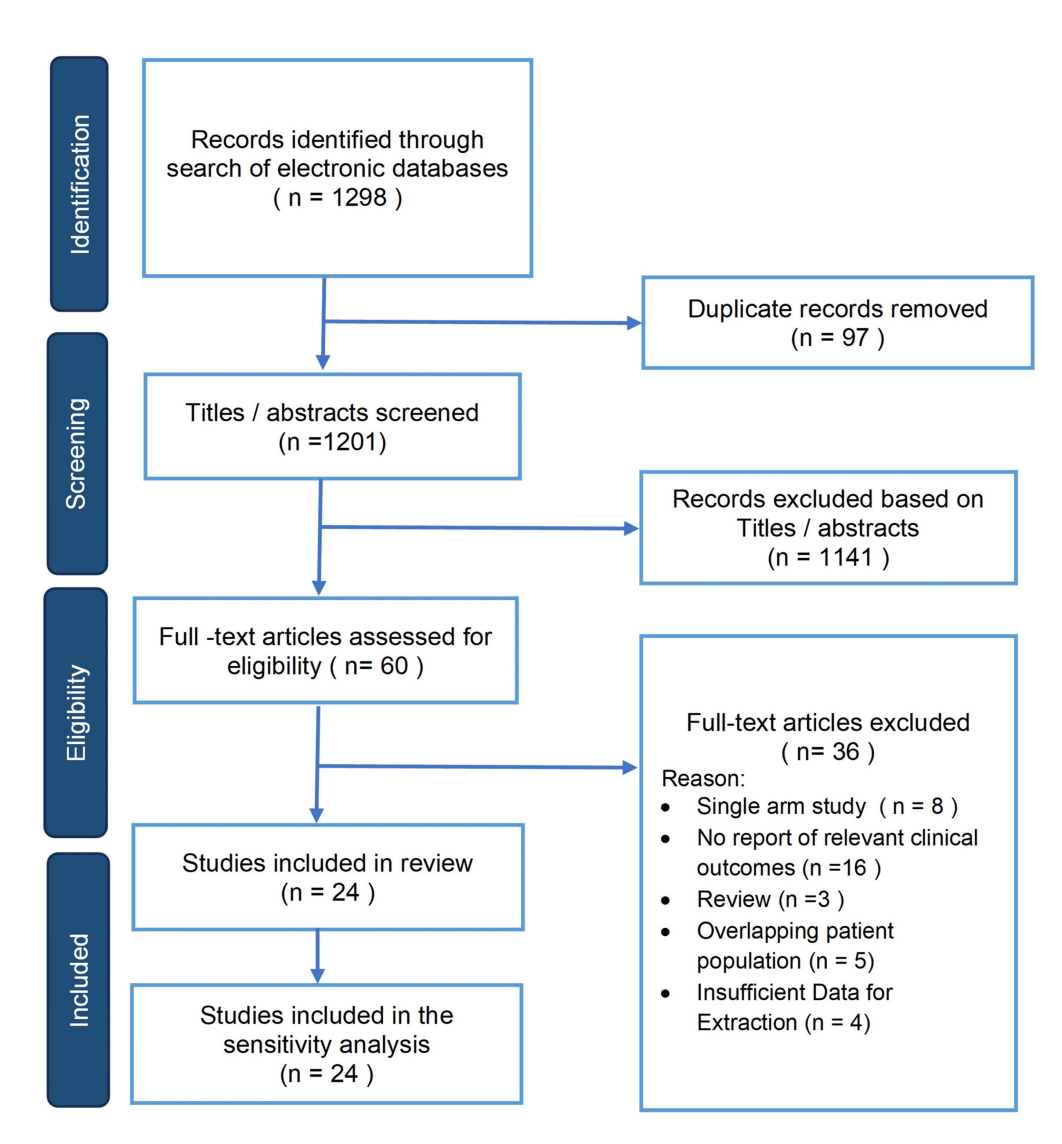
PRISMA flow diagram PRISMA: Preferred Reporting Items for Systematic Reviews and Meta-Analysis

The eligibility criteria, methodological framework, and investigated outcome parameters of this study were defined in advance and documented in a review protocol registered with PROSPERO, the International Prospective Register of Systematic Reviews (Registration Number: CRD420251126297). The methodology followed and adhered to the standards set by the Preferred Reporting Items for Systematic Reviews and Meta-Analyses (PRISMA) guidelines (Figure 1). 

Eligibility Criteria

All comparative studies, including case-control studies, cohort studies, and randomised controlled trials, with a minimum sample size of 10 patients in each group, were considered for inclusion. Studies were required to compare the outcomes of ACLR in smoking and non-smoking patients. Studies that did not directly compare the two groups or investigated only one group were excluded. Furthermore, case reports, case series, reviews, editorials, conference abstracts without sufficient data, and biomechanical, cadaveric, or animal studies were excluded. Articles published in languages other than English were also not considered.

The population of interest included all adult patients (age > 18) of any gender undergoing primary ACLR. The intervention group consisted of patients identified as current smokers at the time of surgery. This group was compared with patients identified as non-smokers.

Outcomes

The primary outcomes were defined as surgical site infections (SSI), venous thromboembolism (VTE), revision ACLR, and ACL graft rupture. Secondary outcomes included the International Knee Documentation Committee (IKDC) score, the Knee Injury and Osteoarthritis Outcome Score (KOOS), the Lysholm Knee Scoring Scale, and all-cause readmission.

Literature Search Strategy 

For this meta-analysis, we developed a comprehensive search methodology according to thesaurus headings, relevant search operators, and database-specific limits within MEDLINE, Web of Science, and EMBASE. Two authors independently carried out the literature search and evaluated clinical trial registries, including http://www.isrctn.com/, http://clinicaltrials.gov/, and http://apps.who.int/trialsearch/, to identify any ongoing or unpublished studies related to the impact of smoking on ACL reconstruction outcomes. Additionally, the reference lists of all included studies were screened for additional potentially eligible articles. The last literature search was carried out on 21 April 2025.

Selection of Studies

Two independent reviewers screened all titles and abstracts found as a result of the search. Where relevance was indicated, the full-texts of relevant articles were obtained and carefully assessed against the predefined eligibility criteria of this review. Studies that met the inclusion criteria were included for the analysis. Any discrepancies arising during this selection phase were resolved through discussion between the two authors. However, if the disagreement persisted, a third author was consulted.

Data Extraction and Management

A standardised electronic data extraction Excel sheet (Microsoft Corporation, Redmond, WA, US) was developed in line with Cochrane guidance for intervention reviews. Two independent reviewers extracted information on study-related data, patient characteristics, and outcomes. Any discrepancies encountered were resolved through discussion with a third author.

Assessment of Risk of Bias

The methodological quality and risk of bias for all included studies were assessed by two independent authors. For this, the Risk Of Bias In Non-randomized Studies - of Interventions, Version 2 (ROBINS-I V2) assessment tool was used. This tool rates seven domains as "Low," "Moderate," or "Serious," with the study's overall score matching its worst domain. The results showed 6 studies with low bias, 15 with moderate, and 3 with serious. The main problem was bias from confounding, which was moderate or serious in 15 studies. In contrast, all 24 studies had low bias in participant selection and outcome measurement. For missing data, 18 studies had low bias, 5 moderate, and 1 serious. Authors resolved disagreements by discussion; if a consensus could not be reached, a third author was consulted for adjudication.

Summary Measures and Synthesis

For the evaluated dichotomous outcomes (e.g., SSI, VTE, and revision ACLR), the odds ratio (OR) with a 95% confidence interval (CI) was used as the summary measure. For the continuous outcomes (e.g., IKDC Score, KOOS Score ), the mean difference (MD) was used as the summary measure. As all reported dichotomous outcomes were adverse events, an OR greater than 1 indicated a higher risk associated with the smoker group, thus favouring the Non-smoking group. Conversely, an OR less than 1 would favour the Smoking group.

Data extracted from the included studies were initially entered by one reviewer into Review Manager 7.12.0 software (The Cochrane Collaboration, 2024, London, UK) for subsequent analysis. The accuracy of this entered data was then independently verified by a second review author. Random-effects modelling was used for analysis. The results of the analysis for each outcome parameter were reported in a forest plot, along with 95% confidence intervals (CIs). The extent of heterogeneity among the studies was assessed using Cochran's Q test (χ2). Inconsistency was quantified by calculating I² and was interpreted according to the following guide: 0-40% suggesting possibly unimportant heterogeneity; 30-60% indicating moderate heterogeneity; and 61-100% representing high heterogeneity. This is because a high I² value suggests that much of the observed variation is due to genuine differences between studies rather than random chance. Therefore, a pooled summary estimate from highly inconsistent studies must be interpreted with caution.

Furthermore, to investigate potential sources of heterogeneity and confirm the robustness of our findings, we planned to perform sensitivity analyses. This could include a specific analysis excluding studies with a particularly high risk of bias. Furthermore, the influence of each study on the overall effect size and heterogeneity was assessed by re-running the analysis, sequentially omitting one study at a time.

Results

Following the literature search, 1298 articles were identified. After the removal of 97 duplicates, 1201 articles were screened based on their titles and abstracts. Of those, 1141 articles were excluded, and 60 articles were shortlisted for full-text assessment for eligibility. After careful evaluation of their full texts, 36 studies were excluded for the following reasons: 16 did not report relevant clinical outcomes, 8 were single-arm studies, 5 had an overlapping patient population, 4 had insufficient data for extraction, and 3 were review articles. Therefore, 24 studies were included in the final review.

Table 1 presents the study-related data and baseline characteristics of the included populations.

**Table 1:** Study-related data and baseline characteristics of the included populations

Author (year)	Country	Journal	Study design	Total	Smoker	Non-smoker	Age	Sex ( Male %)
Kowalchuk et al., 2009 [9]	USA	The Journal of Arthroscopic and Related Surgery	Prospective cohort study	402	32	370	28.4 ± 11.6	52%
Roecker et al., 2022 [11]	USA	Arthroscopy: The Journal of Arthroscopic and Related Surgery	Retrospective cohort study	217,541	29,318	188,223	NR	NR
Yau et al., 2025 [12]	Hong Kong, China	Journal of Experimental Orthopaedics	Retrospective cohort study	495	99	396	28.5 ± 6.9 vs. 26.9 ± 8.5	95% vs. 77%
Andernord et al., 2015 [13]	Sweden	The American Journal of Sports Medicine	Retrospective cohort study	6995	419	6576	26.65	57.6%
Kaeding et al., 2015 [14]	USA	American Journal of Sports Medicine	Prospective cohort study	2423	207	1962	27 ± 11	55%
Karim et al., 2006 [15]	UK	Journal of Bone & Joint Surgery	Prospective cohort study	304	66	238	32.2 vs.33	75.6% vs. 75.3%
Kim et al., 2014 [16]	South Korea	American Journal of Sports Medicine	Retrospective cohort study	487	165	322	31.32 ± 9.27 vs. 30.43 ± 11.21	NR
Kim et al., 2014 [17]	South Korea	Journal of Bone & Joint Surgery	Retrospective cohort study	251	69	158	32.0 ± 11.75 vs. 34.8 ± 3.83	89.9% vs. 79.7%
Ahldén et al., 2012 [18]	Sweden	The American Journal of Sports Medicine	Retrospective cohort study	4466	293	4173	M: 26.2 ± 9.0, F: 29.0 ± 8.4	57.50%
Brophy et al., 2015 [19]	USA	Journal of Bone & Joint Surgery, Am	Prospective multicenter cohort study	2160	210	1950	26.8 ± 11.0	NR
Brophy et al., 2021 [20]	USA	Journal of Orthopaedic Research	Retrospective cohort study	1399	131	1268	27.8 ± 9.9	58%
Bueneman et al., 2024 [21]	Swedish	Knee Surg Sports Traumatol Arthrosc	Retrospective cohort study	26 882	1 288	25 594	27.4 ± 10	58.20%
Cancienne et al., 2016 [22]	USA	American Journal of Sports Medicine	Retrospective cohort study	13358	1659	11699	32.0 ± 11.75 vs. 34.8 ± 3.83	65%
Cox et al., 2014 [23]	USA	American Journal of Sports Medicine	Prospective cohort study	1395	135	1123	17-35	56%
Cvetanovich et al., 2016 [24]	USA	American Journal of Sports Medicine	Retrospective cohort study	4933	NR	NR	NR	NR
Filbay et al., 2017 [25]	Sweden	British Journal of Sports Medicine	Retrospective cohort study	118	30	77	26 ± 5	73%
Holle et al., 2025 [26]	USA	The American Journal of Sports Medicine	Retrospective cohort study	230,422	22,062	207,462	33.6 ± 11.7 vs. 34.7 ± 12.5	48.4% VS. 50.9%
Kawata et al., 2018 [27]	Japan	Knee Surg Sports Traumatol Arthrosc	Retrospective cohort study	30,529	7,504	23,025	NR	NR
Kraus et al., 2021 [28]	Sweden	American Journal of Sports Medicine	Retrospective cohort study	12,169	713	11324	26.8 ± 11.17	57%
Kvist et al., 2014 [29]	Sweden	The Journal of Arthroscopic and Related Surgery	Retrospective cohort study	9,332	579	8753	27 ± 10 vs. 27 ± 10	NR
Marom et al., 2022 [30]	USA	The American Journal of Sports Medicine	Retrospective cohort study	11,451	48	11403	31.0 ± 12.0	56%
Sproul et al., 2023 [31]	USA	Knee Surg Sports Traumatol Arthrosc	Retrospective cohort study	25,622	1 575	24 047	31.1 vs. 29.9	NR
Traven et al., 2021 [32]	USA	Arthroscopy: The Journal of Arthroscopic and Related Surgery	Prospective cohort study	64,165	2406	61759	28.0 ± 2.83	0%
Westermann et al., 2017 [33]	USA	The Journal of Knee Surgery	Retrospective cohort study	6,398	39	6359	32.8 ± 11.0	63%

Methodological Appraisal

**Figure 2:**
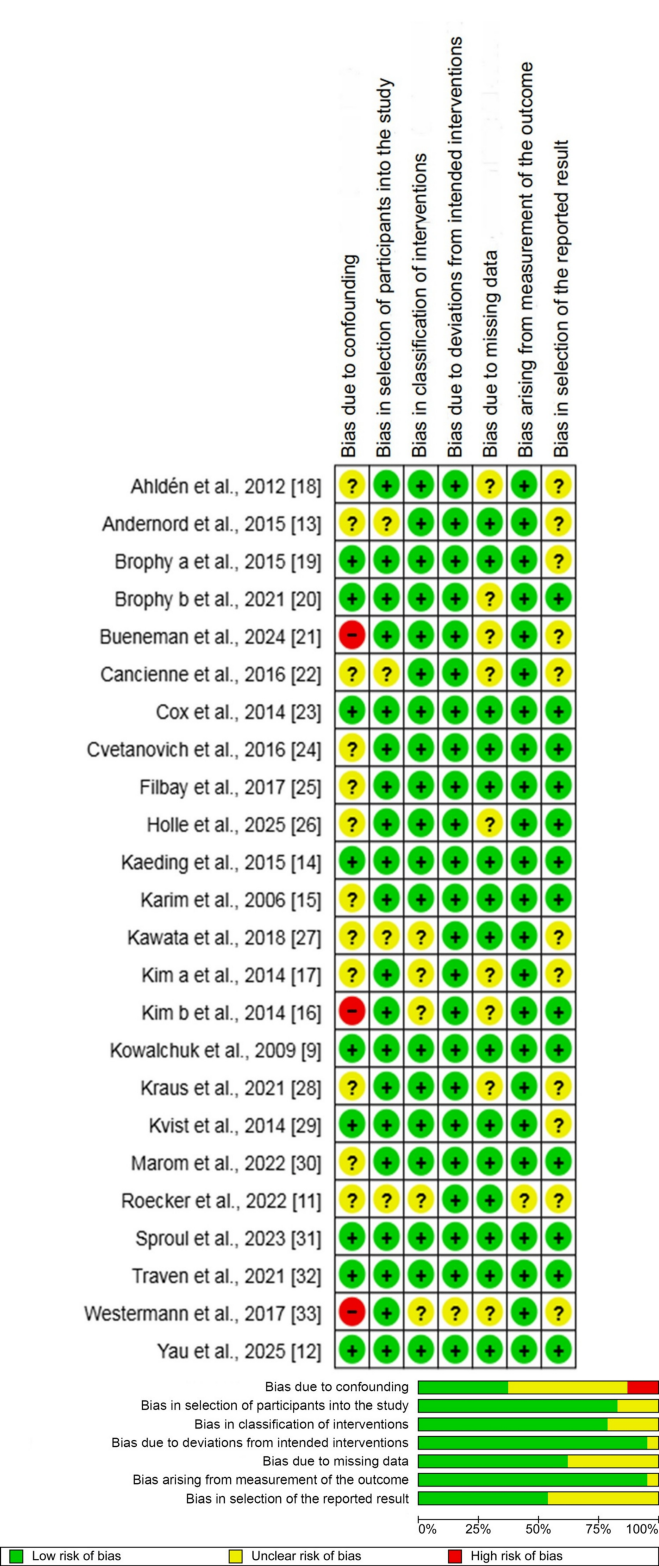
Risk of bias summary and graph showing authors' judgments about each risk of bias item for observational studies.

The methodological quality of the 24 included non-randomised studies was evaluated using the (ROBINS-I V2 assessment tool. The detailed risk of bias assessment is presented in Figure 2 (risk of bias summary and graph).

Outcome Synthesis

The statistical analysis and generation of forest plots for the meta-analysis were conducted using Review Manager (RevMan) (Version 7.12.0) [34].

Surgical Site Infections

**Figure 3:**
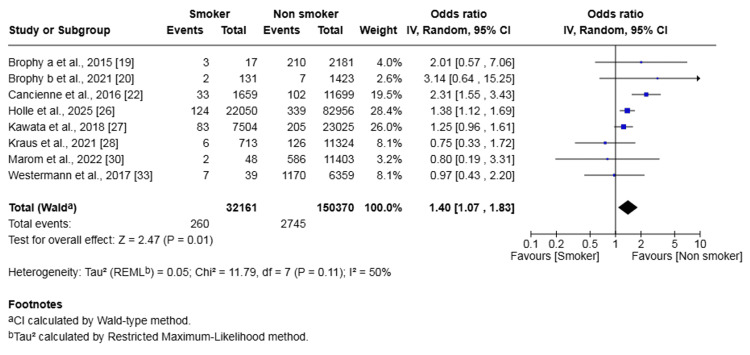
Forest plot of comparison of the outcomes of surgical site infections following anterior cruciate ligament reconstruction in patients who smoked and those who did not The solid squares denote the odds ratio. The horizontal lines represent the 95% confidence intervals (CIs), and the diamond denotes the pooled effect size. MH, Mantel-Haenszel test

Eight studies involving 182,531 patients reported on SSIs. The meta-analysis found that smoking was associated with a significantly higher odds of developing a surgical site infection (OR 1.40, 95% CI (1.07, 1.83), p = 0.01) (Figure 3). Moderate heterogeneity was detected among the studies (I² = 50%, p=0.11).

Venous Thromboembolism (VTE)

**Figure 4:**
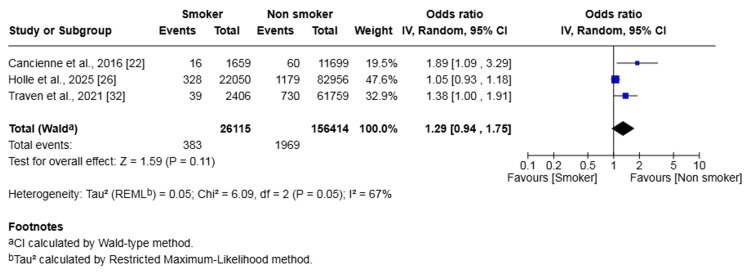
Forest plot of the comparison of outcomes of venous thromboembolism following anterior cruciate ligament reconstruction in patients who smoked versus those who did not The solid squares denote the odds ratio. The horizontal lines represent the 95% confidence intervals (CIs), and the diamond denotes the pooled effect size. MH, Mantel-Haenszel test

Three studies (182,529 patients) reported on VTE. The rate was 1.47% in the smoker group and 1.26% in the non-smoker group. The difference in risk was not statistically significant (OR 1.29, 95% CI (0.94, 1.75), p = 0.11) (Figure 4). Heterogeneity was moderate (I² = 67%, p=0.05).

Revision ACLR

**Figure 5:**
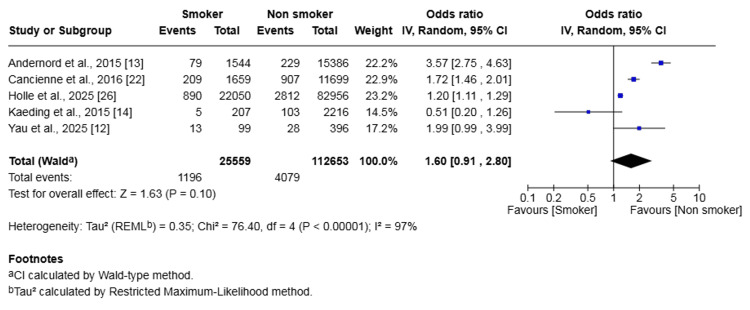
Forest plot of the comparison of outcomes of revision following anterior cruciate ligament reconstruction in patients who smoked and those who did not The solid squares denote the odds ratio. The horizontal lines represent the 95% confidence intervals (CIs), and the diamond denotes the pooled effect size. MH, Mantel-Haenszel test

Five studies (138,212 patients) investigated revision rates. The revision rate was 4.68% in the smoker group versus 3.62% in the non-smoker group. This difference was not statistically significant (OR 1.60, 95% CI (0.91, 2.80), p = 0.10) (Figure 5). There was substantial heterogeneity detected among studies (I² = 97%, p < 0.00001).

ACL Graft Rupture

**Figure 6:**
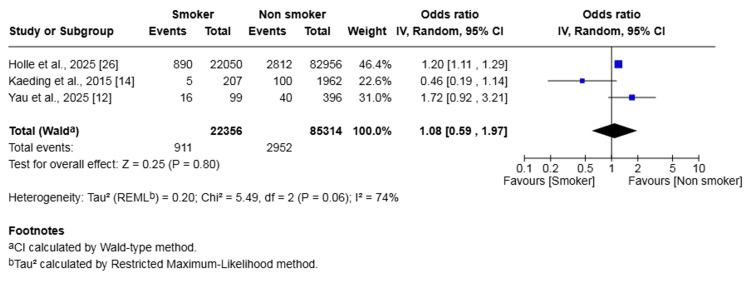
Forest plot of the comparison of outcomes of ACL graft rupture following ACL reconstruction in patients who smoked and those who did not The solid squares denote the odds ratio. The horizontal lines represent the 95% confidence intervals (CIs), and the diamond denotes the pooled effect size. ACL, anterior cruciate ligament; MH, Mantel-Haenszel test

Three studies (107,670 patients) reported on ACL graft rupture. The rate of rupture was 4.07% in the smoker group and 3.46% in the non-smoker group. The difference in risk was not statistically significant (OR 1.08, 95% CI (0.59, 1.97), p = 0.80) (Figure 6). Heterogeneity was moderate (I² = 74%, p=0.06).

IKDC Scores

**Figure 7:**
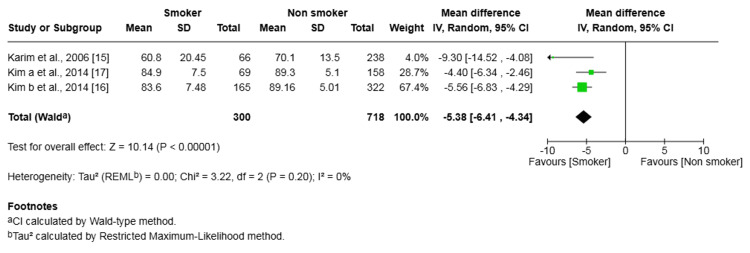
Forest plot of the comparison of outcomes of IKDC scores following anterior cruciate ligament reconstruction in patients who smoked and those who did not The solid squares denote the odds ratio. The horizontal lines represent the 95% confidence intervals (CIs), and the diamond denotes the pooled effect size. IKDC, International Knee Documentation Committee; MH, Mantel-Haenszel test

Three studies (1,018 patients) reported on IKDC scores. The pooled analysis showed significantly lower (worse) scores in the smoker group (mean difference -5.38, 95% CI (-6.41, -4.34), p < 0.00001) (Figure 7). No heterogeneity was found among the studies (I² = 0%, p=0.20).

Lysholm Knee Score

**Figure 8:**
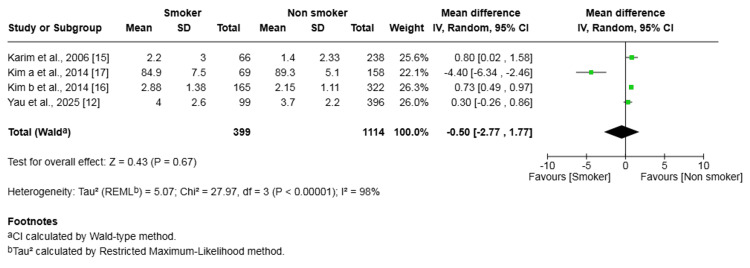
Forest plot of the comparison of outcomes of the Lysholm knee score following anterior cruciate ligament reconstruction in patients who smoked and those who did not The solid squares denote the odds ratio. The horizontal lines represent the 95% confidence intervals (CIs), and the diamond denotes the pooled effect size. MH, Mantel-Haenszel test

Four studies (1,513 patients) investigated the Lysholm Knee Score. The difference in scores between the smoker and non-smoker groups was not statistically significant (mean difference -0.50, 95% CI (-2.77, 1.77), p = 0.67) (Figure 8). There was substantial heterogeneity among studies (I² = 98%, p < 0.00001).

Patient-Reported Outcomes for KOOS

**Figure 9:**
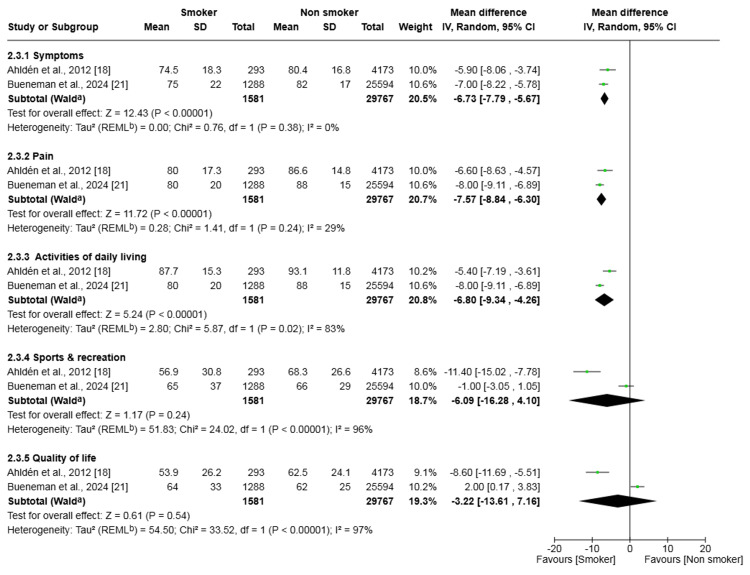
Forest plot of the comparison of outcomes of patient-reported outcomes for KOOS following anterior cruciate ligament reconstruction in patients who smoked and those who did not The solid squares denote the odds ratio. The horizontal lines represent the 95% confidence intervals (CIs), and the diamond denotes the pooled effect size. KOOS: Knee Injury and Osteoarthritis Outcome Score; MH, Mantel-Haenszel test

For the KOOS, two studies (31,348 patients) were analysed across five subscales (Figure 9). Smokers reported significantly worse symptoms (mean difference -6.73, 95% CI (-7.79, -5.67), p < 0.001), with no heterogeneity (I² = 0%, p=0.38), and reported significantly more pain (mean difference -7.57, 95% CI (-8.84, -6.30), p < 0.001), with low heterogeneity (I² = 29%, p=0.24). Furthermore, smokers reported significantly more difficulty with daily living activities (mean difference -6.80, 95% CI (-9.34, -4.26), p < 0.001), with substantial heterogeneity (I² = 83%, p=0.02). In contrast, the difference in sports and recreation scores was not statistically significant (mean difference -6.09, 95% CI (-16.28, 4.10), p = 0.24), with substantial heterogeneity (I² = 96%, p < 0.001). Similarly, the difference in quality of life scores was not statistically significant (mean difference -3.22, 95% CI (-13.61, 7.16), p = 0.54), with substantial heterogeneity (I² = 97%, p < 0.001).

Sensitivity Analysis

The direction of the result stayed the same whether we used the risk ratio (RR) or the risk difference (RD). During the leave-one-out sensitivity analysis, the results for most outcomes remained stable. However, two analyses showed notable changes. In the analysis of revision rate, the removal of the study by Kaeding et al., 2015 [14], changed the pooled effect, making the result significant (OR 1.93, 95% CI 1.19 to 3.13, P=0.008) while heterogeneity remained high (I2=96%). In the analysis of the Lysholm knee score, removal of the study by Kim et al., 2014 [17], made the results significant (MD 0.66, 95% CI 0.40 to 0.91, P<0.001) and substantially reduced heterogeneity (I2=11%).

Discussion

In view of the ongoing uncertainty regarding the impact of smoking on outcomes following ACLR, this comprehensive systematic review and meta-analysis was conducted with the inclusion of 24 comparative studies. The analysis included a total of 672,241 patients, comprising 69,113 in the smoker group and 603,128 in the non-smoker group, to compare postoperative outcomes.

Our meta-analysis revealed that smoking has a significant effect on outcomes following ACLR. Specifically, smoking was associated with a significantly higher risk of SSI. Furthermore, smokers demonstrated clinically and statistically significant poorer PROMs, with lower scores on the IKDC and several subscales of the KOOS, including symptoms, pain, and activities of daily living. In contrast, our analysis did not find a statistically significant difference between smokers and non-smokers in the rates of ACL graft rupture, all-cause revision, VTE, or the Lysholm knee score.

The finding that smoking increases the risk of SSI is well-supported by established pathophysiological mechanisms. Tobacco use directly compromises wound healing on multiple fronts. At a cellular level, nicotine compromises wound healing by reducing blood flow, promoting clot formation, suppressing the immune response (e.g., T-cell function), and impeding the collagen synthesis essential for structural repair [35-38].

While the evidence for smoking's impact on wound healing and infection is clear, its effect on graft integrity appears less definitive. Interestingly, our finding of no significant difference in rupture or revision rates is consistent with several large-scale studies. For instance, Andernord et al., in a prospective study of 16,930 primary ACLRs from the Swedish National Knee Ligament Register, found no significant link between tobacco use and revision surgery [13]. Similarly, Kaeding et al. investigated risk factors in 2,683 patients and reported that smoking status was not associated with graft rupture [14]. A systematic review by Cronström et al. also reported no association, though they noted the quality of the included papers was low. A large body of evidence suggests that the link between smoking and graft failure is complex and may be influenced by numerous patient, surgeon, and disease-related factors [39-43].

The most consistent finding of our analysis was the negative impact of smoking on PROMs. Smokers reported significantly worse IKDC scores and greater issues with symptoms, pain, and daily activities on the KOOS. However, the clinical significance of these statistical findings must be assessed. Our pooled analysis found a mean difference of -5.38 for the IKDC score. This finding is below the commonly accepted minimal clinically important difference (MCID) for the IKDC, which has been established as 8.5 to 8.6 points [44]. Similarly, our findings for KOOS symptoms (-6.73), pain (-7.57), and activities of daily living (ADL) (-6.80) are also below their established MCID thresholds, reported as 8.2-8.4, 9.1-9.3, and 9.0-9.2, respectively [44]. This suggests that while smoking has a consistent, statistically negative impact on these outcomes, the magnitude of this effect may not be large enough to be perceived as clinically meaningful by all patients. This aligns with the work of Karim et al., who suggest that a poorer outcome can be predicted for smokers undergoing this procedure [15]. The inferior functional scores may not be solely due to the knee itself, but could also be a reflection of the poor general health condition often seen in smokers [45]. Moreover, the inferior functional scores related to pain in smokers are rooted in fundamental changes to the pain processing system at the receptor level. Chronic nicotine exposure leads to significant adaptation, including nicotinic acetylcholine receptor (nAChR) desensitisation and other neuronal plastic changes. This process results in a paradoxical upregulation of nAChR expression in the brain, leaving smokers with greater densities of these receptors than non-smokers [46-50].

This study's primary strength lies in its robust sample size, encompassing over 670,000 patients. However, several limitations must be considered when interpreting the findings. First, our analysis is constrained by the nature of the available literature, as all included publications were non-randomised, observational studies. This reliance on observational data introduces an inherent risk of selection bias. Second, a significant limitation of this review is that the included studies grouped all tobacco users together. We were therefore unable to perform subgroup analyses based on smoking type (e.g., cigarette vs. smokeless) or dose (e.g., pack-years), which is a key area for future research. Furthermore, the potential for reporting bias exists, as the social stigma associated with smoking may lead patients to underreport their habits [15-17]. The between-study heterogeneity was observed to be moderate to substantial in the analysis of most outcomes, including ACL graft rupture (I2: 74%), revision rates (I2: 97%), VTE (I2: 67%), SSI (I2: 50%), the Lysholm knee score (I2: 98%), and most KOOS subscales. Conversely, heterogeneity was found to be low or absent in the analyses of IKDC scores (I2: 0%) and the KOOS subscales for symptoms (I2: 0%) and pain (I2: 29%). This high degree of inconsistency may be driven by several factors, such as differences in study populations, varying definitions of 'smoker' (as all tobacco users were aggregated), different surgical techniques, and varying follow-up durations. This variability indicates that the included studies reported diverse results; consequently, the certainty of the evidence for several key outcomes was judged to be moderate to low. Furthermore, the included studies were predominantly from North America and Europe, and future research would benefit from more globally representative data. Finally, the conversion of median and interquartile range (IQR) data to mean and standard deviation using the method described by Hozo et al. may have introduced a statistical bias into our results [51]. This approach, however, was essential for including multiple studies that would have otherwise been excluded from our analysis. The original authors noted that while mean estimates were often fairly accurate, the corresponding variance estimates could be less precise. We therefore acknowledge this potential imprecision in the pooled results for outcomes that required this conversion. 

## Conclusions

Meta-analysis of the best available evidence demonstrated that smoking is associated with a higher risk of SSI following ACLR. The burden on patients was comprehensive, as smokers also demonstrated clinically and statistically significant poorer PROMs on the IKDC and several KOOS subscales, representing a considerable burden on functional recovery. Given the observational nature of the included studies and the noted risk of bias, these findings demonstrate a strong association, but a direct causal relationship cannot be established. We do not hesitate to encourage robust preoperative counselling on smoking cessation to mitigate the clearly elevated risks of postoperative complications and to improve PROMs. Future high-quality research may provide stronger evidence to guide patient optimisation and further refine surgical decision-making.

## Figures and Tables

**Figure 10:**
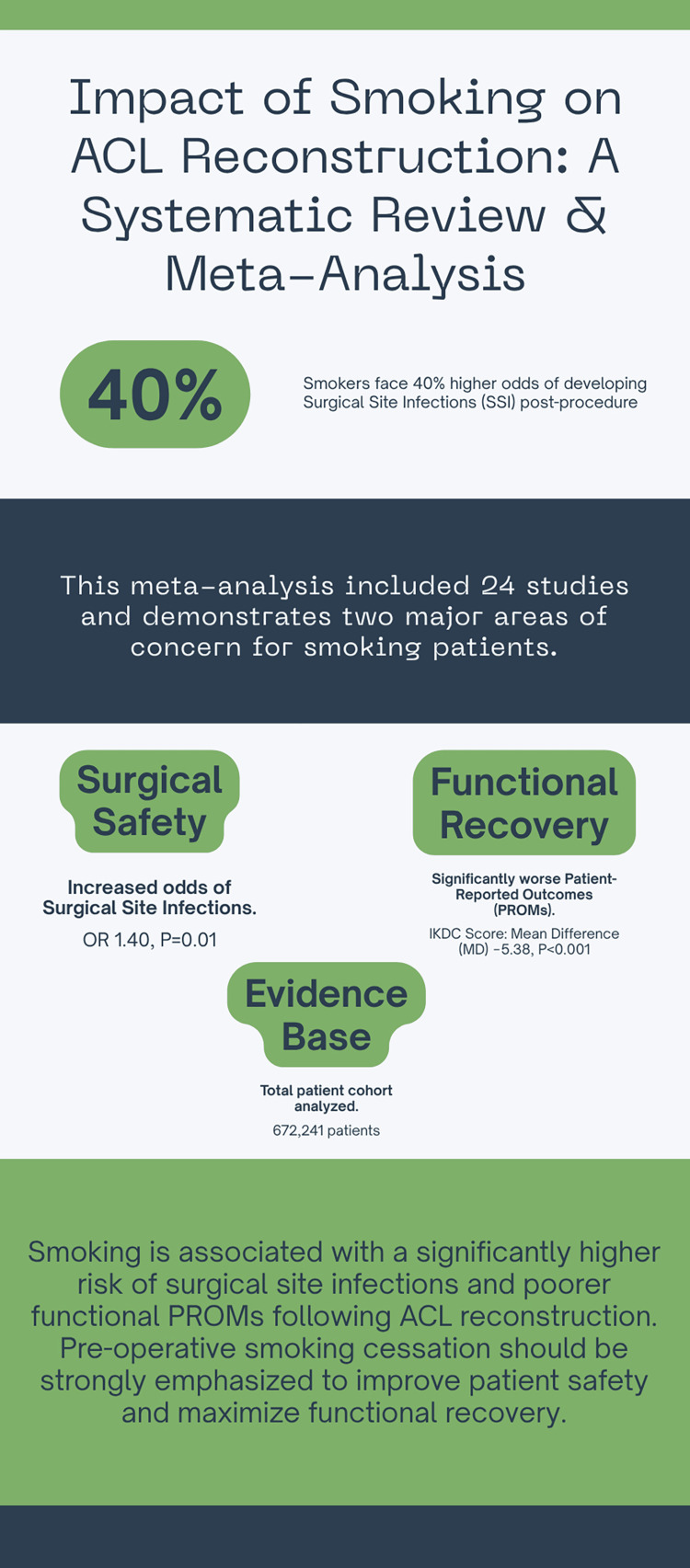
Graphical abstract of the impact of smoking on ACL reconstruction outcomes ACL, anterior cruciate ligament; IKDC: International Knee Documentation
